# Pathogenic Functions of Tumor Necrosis Factor Receptor-**Associated** Factor 6 Signaling **Following** Traumatic Brain Injury

**DOI:** 10.3389/fnmol.2021.629910

**Published:** 2021-04-21

**Authors:** Huan Huang, Anqi Xia, Li Sun, Chun Lu, Ying Liu, Zhenjie Zhu, Siye Wang, Junyan Cai, Xiaoyun Zhou, Su Liu

**Affiliations:** ^1^Department of Rehabilitation Medicine, Affiliated Hospital of Nantong University, Nantong, China; ^2^School of Medicine, Nantong University, Nantong, China; ^3^Department of Rehabilitation Medicine, Shanghai General Hospital, Shanghai Jiao Tong University, Shanghai, China; ^4^Department of Pathology, Affiliated Hospital of Nantong University, Nantong, China

**Keywords:** neuroinflammation, traumatic brain injury, rat model, lipopolysaccharide, TRAF6

## Abstract

Neuroinflammation contributes to delayed (secondary) neurodegeneration following traumatic brain injury (TBI). Tumor necrosis factor receptor-associated factor 6 (TRAF6) signaling may promote post-TBI neuroinflammation, thereby exacerbating secondary injury. This study investigated the pathogenic functions of TRAF6 signaling following TBI *in vivo* and *in vitro*. A rat TBI model was established by air pressure contusion while lipopolysaccharide (LPS) exposure was used to induce inflammatory-like responses in cultured astrocytes. Model rats were examined for cell-specific expression of TRAF6, NF-κB, phosphorylated (p)-NF-κB, MAPKs (ERK, JNK, and p38), p-MAPKs, chemokines (CCL2 and CXCL1), and chemokine receptors (CCR2 and CXCR2) by immunofluorescence, RT-qPCR, western blotting, and ELISA, for apoptosis by TUNEL staining, and spatial cognition by Morris water maze testing. These measurements were compared between TBI model rats receiving intracerebral injections of TRAF6-targeted RNAi vector (AAV9-TRAF6-RNAi), empty vector, MAPK/NF-κB inhibitors, or vehicle. Primary astrocytes were stimulated with LPS following TRAF6 siRNA or control transfection, and NF-κB, MAPKs, chemokine, and chemokine receptor expression levels evaluated by western blotting and ELISA. TRAF6 was expressed mainly in astrocytes and neurons of injured cortex, peaking 3 days post-TBI. Knockdown by AAV9-TRAF6-RNAi improved spatial learning and memory, decreased TUNEL-positive cell number in injured cortex, and downregulated expression levels of p-NF-κB, p-ERK, p-JNK, p-p38, CCL2, CCR2, CXCL1, and CXCR2 post-TBI. Inhibitors of NF-κB, ERK, JNK, and p38 significantly suppressed CCL2, CCR2, CXCL1, and CXCR2 expression following TBI. Furthermore, TRAF6-siRNA inhibited LPS-induced NF-κB, ERK, JNK, p38, CCL2, and CXCL1 upregulation in cultured astrocytes. Targeting TRAF6-MAPKs/NF-κB-chemokine signaling pathways may provide a novel therapeutic approach for reducing post-TBI neuroinflammation and concomitant secondary injury.

## Introduction

Severe traumatic brain injury (sTBI) is a frequent cause of physical disability, cognitive dysfunction, and accidental death. Physical and cognitive deficits result from both primary injury to the trauma site and delayed secondary injury to surrounding structures. Neuroinflammation is a major pathogenic mechanism for secondary brain injury following TBI (Chiu et al., 2016; Simon et al., 2017; Liu et al., 2019), characterized by edema, microglial and astrocytic activation and migration, and the release of inflammatory cytokines and chemokines (Gyoneva and Ransohoff, 2015; Karve et al., 2016; Webster et al., 2017). Thus, inhibition of these processes following sTBI may significantly reduce the progressive deficits associated with secondary brain injury.

Chemokines such as chemokine C-C motif ligand 2 (CCL2) and chemokine C-X-C motif ligand 1 (CXCL1) are critical signaling factors regulating post-TBI neuroinflammation. Cortical levels of these cytokines rise abruptly following injury, with CCL2 demonstrating higher peak levels compared to many other inflammatory mediators in TBI rats (Dalgard et al., 2012). Sustained elevation of CCL2 was detected in the cerebrospinal fluid (CSF) of sTBI patients for 10 days after trauma (Semple et al., 2010). Our preliminary study found that CCL2 was mainly co-localized with the astroglial marker glial fibrillary acidic protein (GFAP), while the CCL2 receptor chemokine C-C motif receptor 2 (CCR2) was mainly co-localized with the neuronal nuclear marker NeuN in TBI model rats, suggesting that CCL2–CCR2 signaling is a major driver of neuroinflammation, neurodegeneration, and concomitant motor and cognitive dysfunction following TBI (Liu et al., 2013a). We further demonstrated that *in vitro* administration of pro-inflammatory lipopolysaccharide (LPS) significantly upregulated expression levels of CCL2 and CXCL1 as well as phosphorylation (activation) of the stress-associated transcription factor nuclear factor-kappa B (NF-κB) and the mitogen-activated protein kinases (MAPKs), c-jun N-terminal kinase (JNK), extracellular signal-regulated kinase (ERK), and p38 in cultured astrocytes prepared from cerebral cortices of neonatal rats. Conversely, neuroinflammation was modulated via regulation of LPS-induced MAPK/NF-κB-CCL2/CXCL1 signaling pathways (Liu et al., 2018).

**FIGURE 1:**
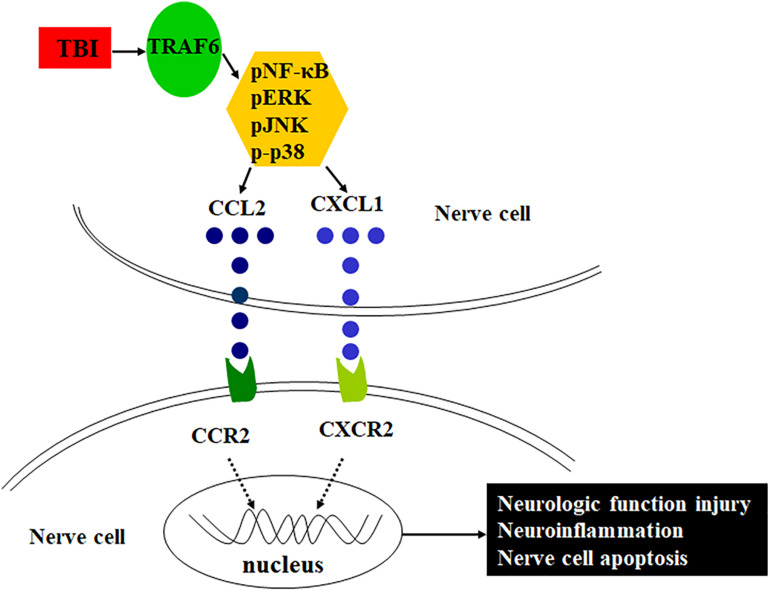
Schematic of the possible mechanism of neuroinflammation following TBI. TRAF6 expression is upregulated after TBI, which activates downstream MAPKs or NF-κB intracellular signaling pathways, induces the expression of chemokines CCL2 and CXCL1, and acts on the corresponding receptors CCR2 and CXCR2, contributes to neuroinflammation, and then leads to pathological changes such as neurologic function injury and nerve cell apoptosis.

Tumor necrosis factor (TNF) receptor-associated factor 6 (TRAF6), a member of the TNF receptor-associated factor family, is a major regulator of central nervous system (CNS) development and neuroinflammation (Aarts et al., 2017; Li et al., 2017) as well as of neural cell apoptosis by directly interacting with the death domain of caspase-8 (Dou et al., 2018). Chen et al. (2011) found that TRAF6 was highly expressed in cortical reactive astrocytes and neurons after TBI and promoted neuronal apoptosis. Other studies have reported that TRAF6 acts as an adaptor in a number of signaling pathways that positively regulate downstream kinase cascades, such as NF-κB, JNK, and p-38, among others (Lu et al., 2014; Zeng et al., 2015; Li et al., 2019). Based on these findings, the present study examined if TRAF6 participates in severe TBI-induced neuroinflammation and associated functional deficits by regulating MAPK/NF-κB-CCL2/CXCL1 signaling pathways. Further, we examined if disruption of these pathways or modulation of these signaling molecules expression can reduce neuroinflammation and functional deficits following severe TBI. Schematic of the possible mechanism of neuroinflammation following TBI is shown in Figure 1.

## Materials and Methods

### Animals and Surgery

Male Sprague–Dawley rats (230–270 g) were purchased from the Experimental Animal Center of Nantong University (Nantong, China) and housed in an animal room under controlled temperature (23 ± 2°C) and a 12-h/12-h light/dark cycle with free access to water and food; rats used for each experiment are shown in Table 1. All experimental procedures were approved by the Experimental Animal Ethics Committee of Nantong University. Severe traumatic brain injury was induced by controlled cortical impact (CCI) using air pressure (Xiong et al., 2013; Chiu et al., 2016), with slight modifications. Briefly, rats were anesthetized with 10% chloral hydrate, fixed in a stereotactic frame, and subjected to right parietal craniotomy with a dental drill (3 mm posterior to bregma, 3 mm from the midline, diameter of 6 mm) to expose the underlying dura mater. Injury was induced using the TBI-0310 impactor device (Precision Systems and Instrumentation, United States) with the following impact parameters: speed 4 m/s, depth 3 mm, and impact time 150 ms. After injury, the skull skin was sterilized with iodophor. The rat was then placed next to a heater until it regained consciousness before return to the home cage. A sham operation group underwent the same surgical procedure but did not receive experimental TBI.

**TABLE 1:** Number of animals used for each experiment.

**Experiment**	**Sham**	**TBI 1 day**	**TBI 3 days**	**TBI 7 days**	**TBI 10 days**
**Experiment 1**					
Immuno fluorescence	3		3		
**Experiment 2**					
TRAF6+Western blot	3	3	3	3	3
**Experiment 3**					
AAV9-TRAF6-RNAi+MWM			10		
AAV9-TRAF6-NC+MWM			10		
MWM	10		10		
**Experiment 4**					
AAV9-TRAF6-RNAi+TUNEL			3		
AAV9-TRAF6-NC+TUNEL			3		
TUNEL	3		3		
**Experiment 5**					
AAV9-TRAF6-RNAi+Western blot			3		
AAV9-TRAF6-NC+Western blot			3		
Western blot	3		3		
**Experiment 6**					
AAV9-TRAF6-RNAi+RT-PCR/ELISA			6		
AAV9-TRAF6-NC+RT-PCR/ELISA			6		
RT-PCR/ELISA	6		6		
**Experiment 7**					
High-dose BAY117082+ ELISA			6		
Low-dose BAY117082+ ELISA			6		
High-dose PD98059+ ELISA			6		
Low-dose PD98059+ ELISA			6		
High-dose SP600125+ ELISA			6		
Low-dose SP600125+ ELISA			6		
High-dose SB203580+ ELISA			6		
Low-dose SB203580+ ELISA			6		
ELISA	6		6		

### Culture of Primary Cortical Astrocytes

Primary astrocyte cultures were prepared from the cerebral cortices of neonatal rats (Gao et al., 2009; Lu et al., 2014; Liu et al., 2018) purchased from the Experimental Animal Center of Nantong University. Bilateral cerebral cortices were isolated and transferred to ice-cold D-Hank’s buffer. The meninges were carefully removed and the remaining tissues dissociated, filtered through nylon mesh (pore size of 100 μm), and collected by centrifugation at ∼3,000 × *g* for 5 min. The cell pellet was dispersed with a pipette and resuspended in medium containing in low-glucose Dulbecco’s Modified Eagle’s Medium (DMEM, Life Technologies, Gibco BRL Division, Grand Island, NY, United States) supplemented with 10% fetal bovine serum (FBS). The cells were then triturated using glass pipettes, filtered through a screen with 10-μm pores, seeded on six-well plates at a density of 2.5 × 10^5^ cells/cm^2^, and cultured for approximately 10 days with medium replacement every 3 days. Dibutyryl cAMP sodium salt (0.15 mM; Sigma–Aldrich, St. Louis, MO, United States) was added to induce morphological and functional differentiation. The cells were used for experiments 3 days after reaching 90% confluence. Prior to experiments, the medium was replaced with Opti-MEM (Life Technologies, Inc-Gibco BRL Division). The cells were incubated with LPS (1 μg/ml; Santa Cruz Biotechnology, Dallas, TX, United States) for 1–6 h and expression levels of inflammatory signaling factors measured by ELISA and western blotting as described below (Liu et al., 2018).

### Adeno-Associated Virus Injection

We constructed an adeno-associated virus serotype 9 (AAV9) vector expressing TRAF6-specific RNAi (AAV9-TRAF6-RNAi) and an empty AAV9 vector (AAV-NC) (1.64E+13 v.g./ml) as a negative control according to the manufacturer’s protocol (Jikai Gene, Shanghai, China). It was worth noting that AAV9-TRAF6-RNAi contained two specific promoters: the astrocyte promoter (1.36E+13 v.g./ml) and the neuronal promoter (1.22E+13 v.g./ml). So we mixed the two viruses with the astrocyte promoter and the neuronal promoter, respectively, in a ratio of 1:1. Male Sprague–Dawley rats (60–70 g) were randomly divided into four groups: (1) sham, (2) TBI, (3) adeno-associated virus negative control (AAV9-NC) with TB, and (4) interference adeno-associated virus (AAV9-TRAF6-RNAi) with TBI. Rats were anesthetized by intraperitoneal injection of 10% chloral hydrate, shaved, sterilized, and fixed on a brain stereotaxic device. The forehead skin was cut along the midline and three holes drilled on the right side of the parietal bone with a dental drill (1.5 mm posterior to bregma, 1.5 mm lateral to the midline; 1.5 mm posterior to bregma, 3 mm lateral to the midline; 3 mm posterior to bregma, 1.5 mm lateral to the midline) through which 2 μl of viral solution was injected per hole using Hamilton microliter syringes. Injection depth was 1.3 mm and injection speed was 0.2 μl/min. The needle was kept in place for 8 min to insure full vector delivery. The TBI model was created as described 4 weeks after virus injection.

### Drugs and Administration

Selective inhibitors of NF-κB, ERK, JNK, and p38 were purchased from Calbiochem (Merck, Darmstadt, Germany) and dissolved in dimethyl sulfoxide (DMSO). One hour after TBI, the injured area was treated with NF-κB inhibitor (BAY117082), ERK inhibitor (PD98059), JNK inhibitor (SP600125), or p38 inhibitor (SB203580) at 25 mg/10 ml (high dose) or 2.5 mg/10 ml (low dose) as indicated. Alternatively, corresponding control (vehicle) subgroups received 10 ml PBS+DMSO. All injections were performed while the rat was fixed to a stereotaxic frame using 10 ml Hamilton microliter syringes. Drugs and solvent were injected at 2 ml/min (requiring about 5 min), and the needle was kept in place for 5 min. Rats received the indicated injections for three consecutive days. After TBI, the surrounding damaged cerebral cortex tissue was collected for analysis of inflammatory factor expression levels by enzyme-linked immunosorbent assay (ELISA).

Astrocytes were transfected with a small interfering (si)RNA against TRAF6 (Guangzhou RiboBio Co., Ltd., Guangzhou, China) or negative control (NC) siRNA for 72 h and then stimulated with LPS. Afterward, the cells were collected for analysis by ELISA or western blotting.

### Immunofluorescence

Rats were anesthetized by intraperitoneal injection of 10% chloral hydrate, perfused through the heart with normal saline until the liver became white, and then perfused with 4% paraformaldehyde. The whole brain was post-fixed overnight in 4% paraformaldehyde, dehydrated in 20 and 30% sucrose solutions, and then coronally cryo-sectioned to 20-μm slices. The slices were first blocked with 1% bovine serum albumin (BSA) for 2 h at room temperature and incubated overnight at 4°C with mouse anti-TRAF6 monoclonal antibody (sc-8409, 1:200, Santa Cruz Biotechnology) plus the astrocyte marker rabbit anti-GFAP monoclonal antibody (ab7260, 1:500, abcam), the microglial marker goat anti-IBA-1 monoclonal antibody (ab5076,1:500, abcam), or the neuronal marker rabbit anti-NeuN monoclonal antibody (ab177487, 1:500, abcam). Slices were then incubated at room temperature with Cy3-conjugated and Alexa 488-conjugated secondary antibodies (1:1000, Jackson ImmunoResearch, West Grove, PA, United States) for 2 h. The stained sections were examined with a Nikon Ni-E microscope at 20× magnification. Images were analyzed by ImageJ software (NIH).

### Morris Water Maze Test

Three days before TBI modeling, the rats received successive adaptive training trials in the Morris water maze to eliminate the influences of vision and motor function on performance. Place navigation learning was tested starting on the third day after TBI, and the spatial probe trial for spatial memory was conducted 24 h later (Liu et al., 2013b; Tucker et al., 2018). Escape latency to the platform was measured on each of four daily learning trials and averaged. We also recorded swim paths during each trial. If the rat did not reach the platform within 120 s, it was guided to the platform and allowed to stay for 30 s. The escape latency in such cases was recorded as 120 s. The probe trial examined spatial memory for the original platform location by recording the number of crossings over the former platform location after release from the quadrant opposite the target quadrant. Two probe trials were conducted and the average recorded for analysis.

### TUNEL

Terminal deoxynucleotidyl transferase-mediated dUTP nick-end labeling (TUNEL) staining was performed to detect DNA fragmentation as an index of programmed cell death in brain tissues using an apoptosis detection kit (A113, Vazyme, Nanjing, China) according to the manufacturer’s protocol. The red fluorescence of fluorescein-12-dUTP was detected against the blue background of 4′,6-diamidino-2-phenylindole (DAPI) under a fluorescence microscope at 20× magnification. The TUNEL-positive cells in the injured cortex were identified and counted by ImageJ software (NIH).

### Real-Time Quantitative Polymerase Chain Reaction

Rats were anesthetized with 10% chloral hydrate and then perfused via the heart with PBS until the liver turned white. The cerebral cortex around the injured area was excised and total RNA extracted using TRIzol. The RNAs were then reversed transcribed to cDNA. Real-time PCR was performed in a Step One Plus real-time qPCR instrument using the primer sequences shown in Table 2 and the following PCR amplification conditions: pre-denaturation at 95°C for 3 min; 40 cycles of 95°C for 10 s and 60°C for 30 s; dissolution at 95°C for 15 s, 60°C for 60 s, and 95°C for 15 s. Expression of GAPDH served as the internal control and gene expression level was determined using the 2^–Δ^ ^Δ^ ^*CT*^ method.

**TABLE 2:** Primer sequences.

**Genes**	**Primers**	**Sequences (5′–3′)**
GAPDH	Forward	TCCTACCCCCAATGTATCCG
	Reverse	CCTTTAGTGGGCCCTCGG
CCL2	Forward	TGCTGCTACTCATTCACTGGC
	Reverse	CCTTATTGGGGTCAGCACAG
CCR2	Forward	TGCTACTCAGGAATCCTCCACAC
	Reverse	GGCCTGGTCTAAGTGCATGTCAAC
CXCL1	Forward	GCACCCAAACCGAAGTCATA
	Reverse	GGGGACACCCTTTAGCATCT
CXCR2	Forward	TGGTCCTCGTCTTCCTGCTCTG
	Reverse	CGTTCTGGCGTTCACAGGTCTC

### Western Blot

Western blotting was performed according to the previous study (Liu et al., 2018). The antibodies used were shown as follows: TRAF6 (sc-8409,1:500, Santa Cruz), p-NF-κB (3033, 1:1000, Cell Signaling, Danvers, MA, United States), p-ERK (9101, 1:1000, Cell Signaling), p-JNK (4688,1:1000, Cell Signaling), p-p38 (9211,1:1000, Cell Signaling), and GAPDH (MAB374,1:10,000, Millipore, Billerica, MA, United States).

### ELISA

Cell proteins were prepared as described for western blotting and added at 100 μg/well to 96-well plates prepared for ELISA according to the kit manufacturers’ instructions (below). The absorbance of each well at 450 nm was measured and target protein concentrations calculated according to standard curves prepared by the dilution of standards. A rat CXCL1 ELISA kit was purchased from Hangzhou MultiSciences (Lianke) Biotech (EK396/2-96, Hangzhou, Zhejiang, China), a rat CCL2 ELISA kit from R&D Systems (MJE00, Minneapolis, MN, United States), a rat CCR2 ELISA kit from CUSABIO TECHNOLOGY (CSB-EL004841RA, Wuhan, Hubei, China), and a rat CXCR2 ELISA kit from Cloud-Clone Corp (SEC006Ra, Katy, TX, United States).

### Statistical Analysis

All measurement data are expressed as mean ± SEM. Multiple group means were compared by one-way ANOVA with post hoc Bonferroni correction. A *P* < 0.05 (two-tailed) was considered significant for all tests. All statistical analyses were conducted using GraphPad Prism 5.0 (San Diego, CA, United States).

## Results

### Co-localization of TRAF6 With Neuron and Astrocyte Markers in Cerebral Cortex of Traumatic Brain Injury (TBI) Model Rats

**FIGURE 2:**
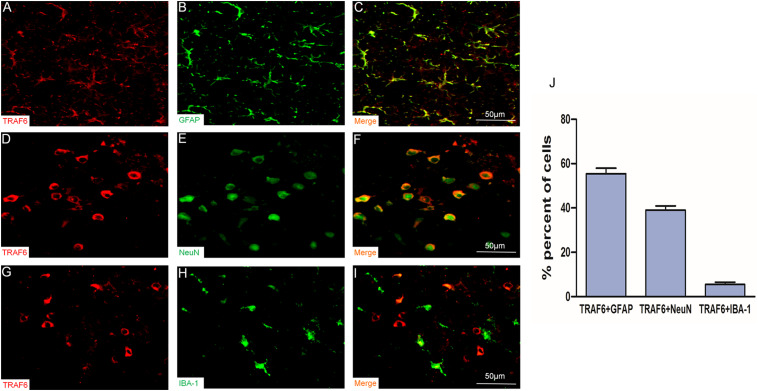
Upregulation of TRAF6 in neurons and astrocytes at the injury site following experimental TBI in rats. TRAF6 (red), GFAP, NeuN and IBA-1 (green), and merged showing the specific cell types expressing TRAF6 **(A–I)**. TRAF6 was co-localized mainly with the astrocytic marker GFAP **(A–C)**, less frequently with the neuronal marker NeuN **(D–F)**, and rarely with the microglial marker IBA-1 **(G–I)**. **(J)** Quantification of co-expression showing that TRAF6 was upregulated mainly in cells positive for the astrocytic marker GFAP and the neuronal marker NeuN.

To determine the specific cell types expressing TRAF6, we performed double immunofluorescence staining using anti-TRAF6 and a second cell-specific marker (GFAP, NeuN, or IBA-1). TRAF6 mainly co-localized with the astrocyte marker GFAP (Figures 2A–C) and the neuronal marker NeuN (Figures 2D–F) but demonstrated almost no co-localization with the microglial marker IBA-1 (Figures 2G–I). According to manual cell counting, 55 ± 3% of astrocytes, 39 ± 2% of neurons, and 6 ± 1% of microglia expressed detectable TRAF6 (Figure 2J).

### TBI Induced Region-Specific Upregulation of TRAF6 Expression in Cerebral Cortex

**FIGURE 3:**
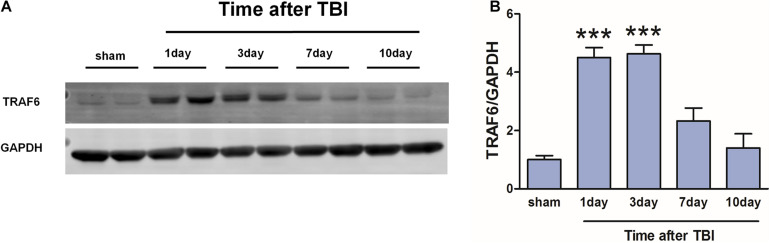
Transiently increased cortical expression of TRAF6 following TBI. **(A)** Protein expression of TRAF6 was determined by Western blot analysis. GAPDH was used as an internal control. **(B)** The optical density of the bands was analyzed by ImageJ. The values are presented as the mean ± SEM. ***P < 0.001 vs. sham group.

Western blot was used to detect changes in cortical TRAF6 expression at 1, 3, 7, and 10 days post-TBI (Figure 3). On day 1 post-TBI, TRAF6 expression was significantly greater in the injured area of the TBI group compared to the sham group. Expression reached a peak at 3 days post-TBI and then gradually declined.

### AAV9-TRAF6-RNAi Injection Rescued Spatial Cognition

**FIGURE 4:**
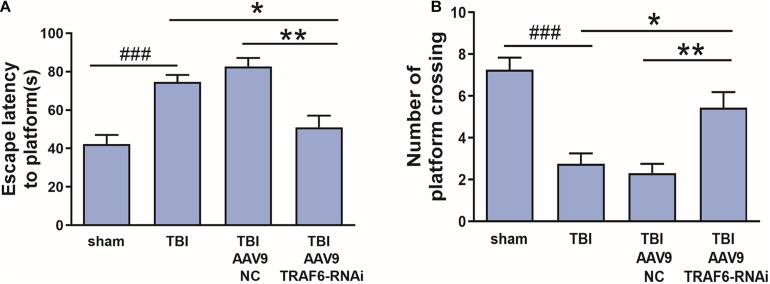
Knockdown of TRAF6 after TBI rescued spatial learning and memory. Sham and TBI model rats receiving intracerebral injection of knockdown vector (AAV9-TRAF6-RNAi) or negative control vector (AAV9-NC) were compared for spatial learning and memory in the Morris water maze at 3 days post-TBI (or sham surgery). **(A)** Escape latency is presented as mean ± SEM (s). Administration of AAV9-TRAF6-RNAi reduced average latency to the escape platform, indicating improved spatial learning following TBI. *P < 0.05 and **P < 0.01 vs. AAV9-TRAF6-RNAi group. ^###^*P* < 0.001 vs. sham group. **(B)** The number of platform location crossings is presented as mean ± SEM. Administration of AAV9-TRAF6-RNAi increased the number of platform crossings following TBI, indicating improved spatial memory. *P < 0.05 and **P < 0.01 vs. AAV9-TRAF6-RNAi group. ^###^*P* < 0.001 vs. sham group.

To evaluate whether TRAF6 signaling pathways contribute to cognitive dysfunction after TBI, rat subgroups receiving Sham, TBI, AAV9-NC+TBI, or AAV9-TRAF6-RNAi+TBI were compared for spatial learning and memory in the Morris water maze. Cognitive functions were significantly decreased after brain injury in the TBI group which compared with sham group. The average latency to the hidden platform during learning trials was significantly lower in the AAV9-TRAF6-RNAi+TBI group compared to TBI and AAV9-NC+TBI groups (Figure 4A), while in the probe trial, the mean number of platform location crossing was significantly greater in the AAV9-TRAF6-RNAi group (Figure 4B). Thus, suppression of the post-TBI increase in TRAF6 partially rescued spatial learning and memory.

### AAV9-TRAF6-RNAi Injection Reduced Nerve Cell Neuronal Apoptosis After TBI

**FIGURE 5:**
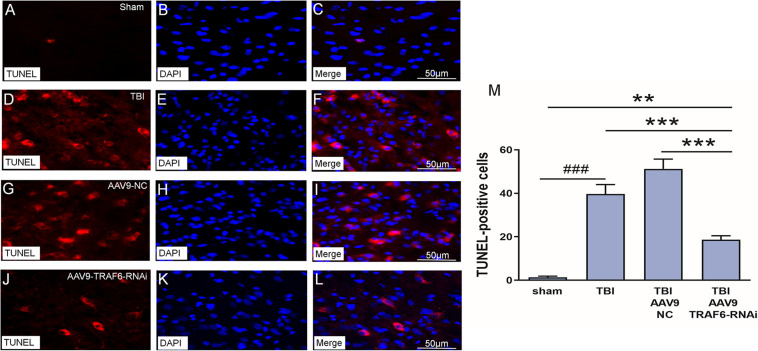
Knockdown of TRAF6 reduced apoptotic death rate following TBI. TUNEL (red), DAPI (blue), and TUNEL+DAPI (merged) images showing apoptotic nerve cell. **(A–C)** Sham-treated rats. **(D–F)** TBI model rats. **(G–I)** TBI model rats injected with AAV9-NC. **(J–L)** TBI model rats injected with AAV9-TRAF6-RNAi. Scale bars are equal to 50 μm. **(M)** AAV9-TRAF6-RNAi treatment decreased nerve cell apoptosis. Values are presented as mean ± SEM. **P < 0.01 and ***P < 0.001 vs. AAV9-TRAF6-RNAi group. ^###^*P* < 0.001 vs. sham group.

We used TUNEL staining of cortical sections to examine the effect of TBI-induced TRAF6 signaling on apoptosis, a major cell death pathway contributing to secondary injury. The number of TUNEL positive (apoptotic) cells was significantly higher in the TBI group compared to that of the sham group. Cell counting revealed a significant decrease in TUNEL-positive cells 3 days post-TBI in TBI model rats receiving AAV9-TRAF6-RNAi compared to the AAV9-NC-treated TBI group (Figure 5).

### AAV9-TRAF6-RNAi Downregulated the Expression Levels of p-NF-κB, p-ERK, p-JNK, and p-p38 in Injured Cortex

**FIGURE 6:**
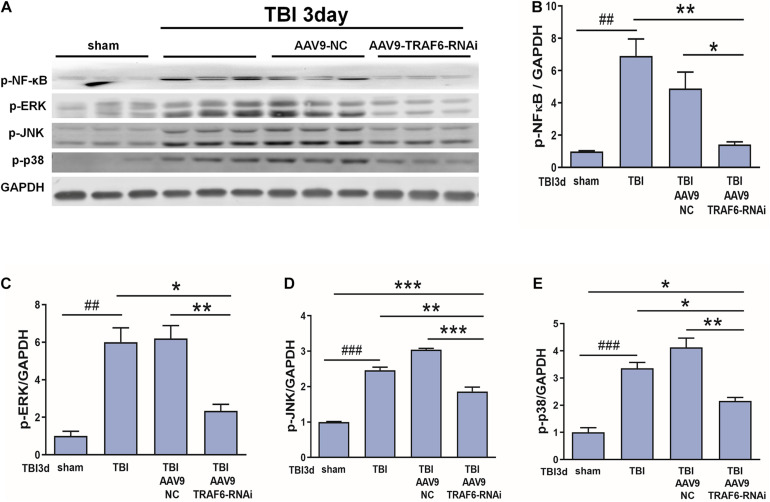
TRAF6 knockdown reduced p-NF-κB, p-ERK, p-JNK, and p-p38 expression in the injured cortex after TBI. **(A)** Western blot analysis was used to detect the expression of p-NF-κB, p-ERK, p-JNK, and p-p38. GAPDH was used as an internal control. **(B–E)** The optical density of the bands was analyzed by ImageJ. *P < 0.05, **P < 0.01, and ***P < 0.001 vs. AAV9-TRAF6-RNAi group. ^##^*P* < 0.01 and ^###^*P* < 0.001 vs. sham group.

Based on the above results and our previous research (Lu et al., 2014; Liu et al., 2018), we speculated that p-NF-κB, p-ERK, p-JNK, and p-p38 may be TRAF6-activated downstream signaling factors contributing to secondary degeneration following TBI. To test this hypothesis, we compared the expression changes in p-NF-κB, p-ERK, p-JNK, and p-p38 among TBI model rats receiving AAV9-TRAF6-RNAi or AAV9-NC pretreatment. Consistent with activation of these signaling pathways by TRAF6, the results showed that the TBI increased the production of p-NF-κB, p-ERK, p-JNK, and p-p38 compared to the sham group, the expression levels of p-NF-κB, p-ERK, p-JNK, and p-p38 at the site of injury were significantly lower in the AAV9-TRAF6-RNAi+TBI group compared to the AAV9-NC+TBI group on day 3 post-TBI (Figure 6).

### AAV9-TRAF6-RNAi Downregulated mRNA and Protein Expression Levels of CCL2, CCR2, CXCL1, and CXCR2 in Injured Cortex

**FIGURE 7:**
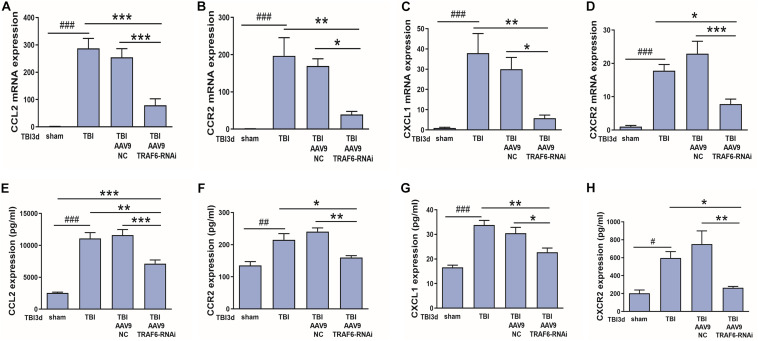
TRAF6 knockdown suppressed CCL2, CCR2, CXCL1, and CXCR2 expression at both mRNA and protein levels in injured cortex after TBI. **(A–D)** AAV9-TRAF6-RNAi downregulated **(A,B)** CCL2 and CCR2 mRNA, **(C,D)** CXCL1 and CXCR2 mRNA, **(E,F)** CCL2 and CCR2 protein, and **(G,H)** CXCL1 and CXCR2 protein expression in injured cortex on day 3 post-TBI. *P < 0.05, **P < 0.01, and ***P < 0.001 vs. AAV9-TRAF6-RNAi group.^ #^*P* < 0.05, ^##^*P* < 0.01, and ^###^*P* < 0.001 vs. sham group.

We also speculated that CCL2 and CXCL1 may be downstream effectors of TRAF6-MAPK-NF-κB signaling. To test this hypothesis, we compared mRNA and protein expression levels of CCL2, CXCL1, CCR2, and CXCR2 between TBI model rats receiving AAV9-TRAF6-RNAi or AAV9-NC pretreatment (Figure 7). Indeed, consistent with regulation by TRAF6, AAV9-TRAF6-RNAi downregulate the mRNA expression levels (Figures 7A–D) and protein expression levels (Figures 7E–H) of CCL2, CCR2, CXCL1, and CXCR2, which had been induced by TBI for 3 days.

### NF-κB, ERK, JNK, and p38 Inhibitors Also Reduced Protein Expression Levels of CCL2, CCR2, CXCL1, and CXCR2 in Injured Cortex

**FIGURE 8:**
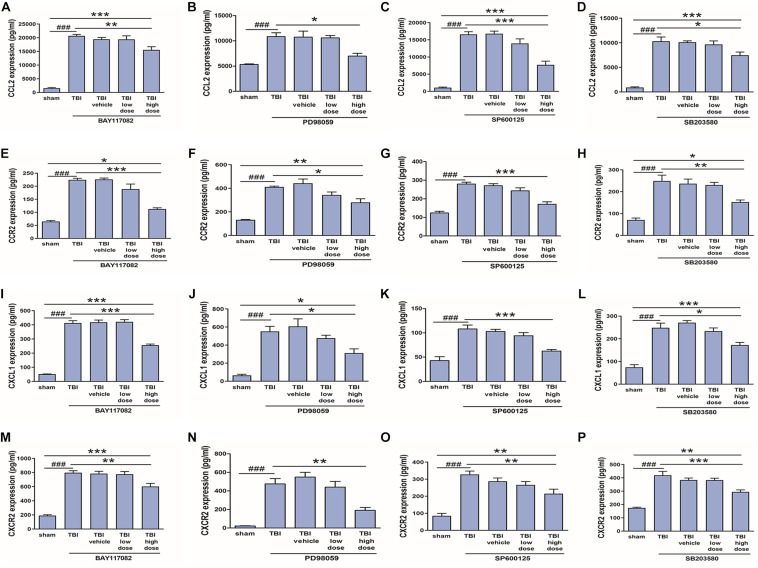
Inhibitors of p-NF-κB and MAPKs reduced upregulation of CCL2, CCR2, CXCL1, and CXCR2 following TBI. A 25 mg/10 ml dose of the p-NF-κB inhibitor BAY117082, p-JNK inhibitor PD98059, p-ERK inhibitor SP600125, or p-p38 inhibitor SB203580 reduced CCL2 **(A–D)**, CCR2 **(E–H)**, CXCL1 **(I–L)**, and CXCR2 **(M–P)** protein expression in the injured cortex as measured by ELISA, while lower doses (2.5 mg/10 ml) had no significant effect. *P < 0.05, **P < 0.01, and ***P < 0.001 vs. high dose group. ^###^*P* < 0.001 vs. sham group.

In our previous study (Liu et al., 2018), we demonstrated that NF-κB, ERK, and JNK inhibitors reduced CCL2 and CXCL1 expression in activated astrocytes. To verify these effects *in vivo*, we measured changes in CCL2, CXCL1, CCR2, and CXCR2 protein expression levels among model rats treated with low (2.5 mg/10 ml) or high (25 mg/10 ml) doses of NF-κB, ERK, JNK, and p38 inhibitors. Indeed, a high dose of the NF-κB inhibitor BAY117082, ERK inhibitor PD98059, JNK inhibitor SP600125, or p38 inhibitor SB203580 downregulates expression of CCL2, CCR2, CXCL1, and CXCR2 at the protein level (Figures 8A–P), which had been induced by TBI for 3 days. Thus, TRAF6-MAPK/NF-κB signaling pathways appear to mediate upregulation of CCL2, CCR2, CXCL1, and CXCR2 following TBI.

### LPS Induced TRAF6 Upregulation in Cultured Astrocytes

**FIGURE 9:**
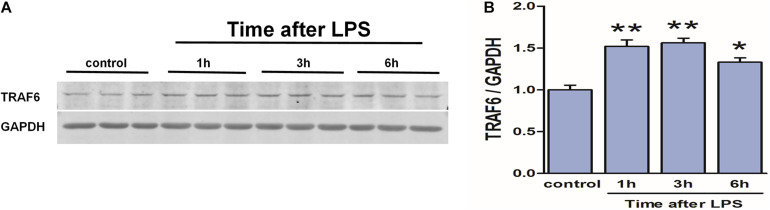
Upregulation of TRAF6 in primary cortical astrocytes in response to LPS. LPS exposure (1 μg/ml) rapidly upregulated TRAF6 protein expression in cultured primary rat astrocytes. **(A)** Western blot analysis was used to detect the expression of TRAF6. GAPDH was used as an internal control. **(B)** The optical density of the bands was analyzed by ImageJ. Values are presented as the mean ± SEM. *P < 0.05 and **P < 0.01 vs. control group.

To verify the link between neuroinflammation and TRAF6 upregulation in astrocytes (the predominant cell type showing TRAF6 upregulation after TBI, Figure 2), we measured TRAF6 protein expression changes in primary astrocytes at 1, 3, and 6 h following stimulation (activation) by the inflammatory inducer LPS (Figure 9). Expression levels of TRAF6 in primary astrocytes were higher at 1 h post-LPS compared to the sham-treated control group and peaked at 3 h before gradually decreasing.

### TRAF6 Knockdown Suppressed p-NF-κB, p-JNK, p-ERK, and p-p38 Expression in LPS-Treated Cultured Astrocytes

**FIGURE 10:**
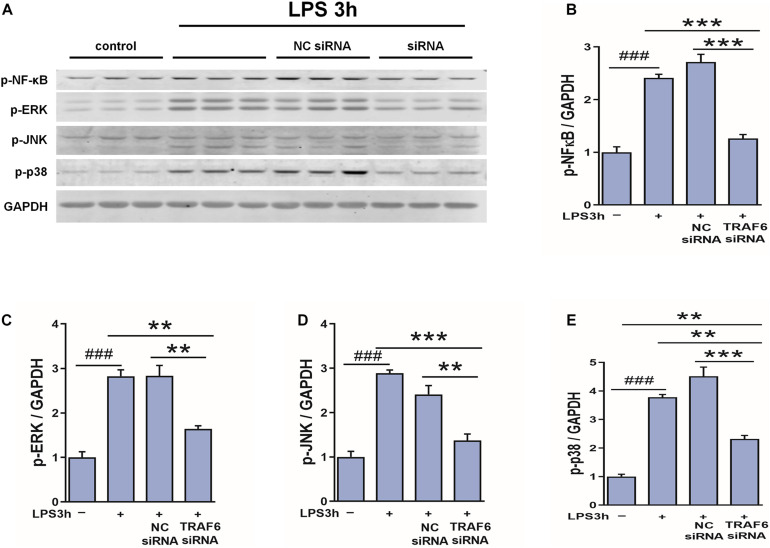
TRAF6 knockdown partially suppressed LSP-induced upregulation of p-NF-κB, p-JNK, p-ERK, and p-p38 in cultured astrocytes. **(A)** Western blot analysis was used to detect the expression of p-NF-κB, p-ERK, p-JNK, and p-p38. GAPDH was used as an internal control. **(B–E)** The optical density of the bands was analyzed by ImageJ. **P < 0.01 and ***P < 0.001 vs. TRAF6 siRNA group.^ ###^*P* < 0.001 vs. control group.

To examine if TRAF6 upregulation in astrocytes activates NF-κB, JNK, ERK, and p38 as downstream effectors, we compared LPS-induced changes in the expression levels of the phosphorylated forms between control astrocytes and astrocytes transfected with TRAF6 siRNA (Figure 10). Indeed, TRAF6 siRNA significantly suppressed LPS-induced upregulation of p-NF-κB, p-JNK, p-ERK, and p-p38 at 3 h post-stimulation. Collectively, these findings (Figures 9, 10) suggest that MAPK/NF-κB signaling pathways are induced in activated astrocytes at the site of TBI via upregulation of TRAF6.

### TRAF6 Knockdown Suppressed CCL2 and CXCL1 Expression in LPS-Treated Astrocytes

**FIGURE 11:**
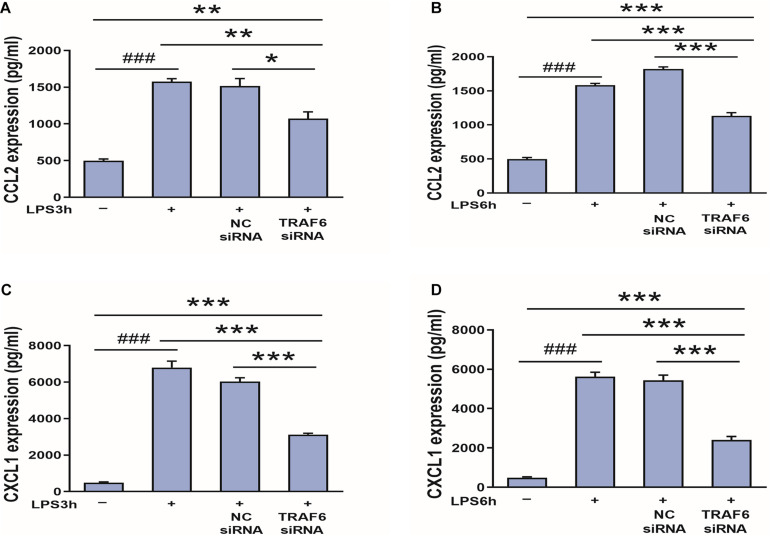
TRAF6 knockdown downregulates CCL2 and CXCL1 expression in LPS-activated astrocytes. **(A,B)** TRAF6 siRNA downregulated CCL2 expression. *P < 0.05, **P < 0.01, and ***P < 0.001 vs. TRAF6 siRNA group. ^###^*P* < 0.001 vs. control group. **(C,D)** TRAF6 siRNA downregulated CXCL1 expression. ***P < 0.001 vs. TRAF6 siRNA group. ^###^*P* < 0.001 vs. control group.

Finally, to examine if CCL2 and CXCL1 are downstream effectors of TRAF6, expression levels were compared between control astrocyte cultures and cultures pretreated with TRAF6 siRNA. As shown in Figure 11, TRAF6 siRNA significantly reduced CCL2 and CXCL1 expression levels in astrocytes treated for 3 or 6 h with LPS treatment.

## Discussion

Tumor necrosis factor receptor-associated factor 6 acts as a critical regulator of acquired immunity by binding to TNF super family and Toll/interleukin-1 receptor super family members. Due to the large number of binding partners, TRAF6 can exert complex regulation of inflammatory signaling pathways. Numerous studies have reported TRAF6 upregulation associated with CNS damage due to TBI, stroke, neurodegenerative diseases, and neuropathies (Chen et al., 2011, 2019; Aarts et al., 2017; Dou et al., 2018; Wu et al., 2018). In the present study, we found that TRAF6 was mainly expressed in astrocytes and neurons of injured cortex and that the expression level of TRAF6 reached its peak in the injured cerebral cortex 3 days following experimental TBI. TRAF6 knockdown also reduced neuronal apoptosis and rescued spatial learning and memory at 3 days after TBI. Therefore, TRAF6 may play an important role in secondary neuroinflammatory injury in the early stages after TBI.

Traumatic brain injury in rats significantly upregulated the local expression levels of NF-κB, MAPKs (ERK, JNK, and p38), and other signaling molecules implicated in the neuroinflammatory response of rats following TBI (Chu et al., 2013; Kim et al., 2015; Marshall et al., 2017; Yang et al., 2017). Suppression of these inflammatory signaling pathways may thus reduce secondary injury from TBI (Geng et al., 2016; Meng et al., 2016; Qian et al., 2017; Kong et al., 2019). In this study, we used AAV9-TRAF6-RNAi to interfere with the expression of TRAF6 and found that knockdown significantly reduced the upregulation of p-NF-κB, p-JNK, p-ERK, p-p38, CCL2, CCR2, CXCL1, and CXCR2 expression levels observed after TBI, indicating that TRAF6 could regulate these signal molecules. We further found that application of inhibitors of NF-κB, p38, ERK, and JNK decreased the upregulated expression of CCL2, CCR2, CXCL1, and CXCR2 following TBI, showing that NF-κB, p38, ERK, and JNK could regulate CCL2, CCR2, CXCL1, and CXCR2. Collectively, these results indicate that TRAF6 upregulation in astrocytes and neurons after TBI induces neurologic dysfunction and apoptosis by activating NF-κB/MAPK-CCL2/CXCL1 signaling pathways, while AAV9-TRAF6-RNAi likely alleviated post-TBI neuroinflammation by targeting NF-κB/ERK/JNK/p38-CCL2/CXCL1 signaling pathways.

Astrocytes and microglia are the major immune cells of the brain parenchyma that mediate inflammation. We had verified that downstream signal molecule of TRAF6 including CCL2 and CXCL1 was mainly expressed in astrocytes of injured cortex after TBI in our previous study *in vivo*. Our previous study further showed that LPS significantly upregulated CCL2, CXCL1, p-NF-κB, p-ERK, p-JNK, and p-p38 in cultured astrocytes *in vitro*, and that inhibitors of NF-κB, ERK, and JNK partially suppressed CCL2 and CXCL1 overexpression induced by LPS. Alternatively, the p38 inhibitor had no obvious effect on expression levels of CCL2 and CXCL1. We further demonstrated that reducing neuroinflammation via downregulation of LPS-induced MAPKs (ERK and JNK)/NF-κB-CCL2/CXCL1 signaling pathways in astrocytes (Liu et al., 2018). Results of the study indicated that LPS induced significant upregulation of TRAF6 in primary cultured astrocytes and the peak expression level was reached after 3 h of LPS treatment. Based on our preliminary data, astrocytes were transfected with a small interfering siRNA against TRAF6 in this study. We found that a TRAF6 siRNA downregulated the expression levels of CCL2, CXCL1, p-NF-κB, p-ERK, p-JNK, and p-p38 in cultured astrocytes activated by LPS. Taken together, these results suggest that TRAF6 can modulate neuroinflammation via regulation of the LPS-induced-MAPKs (ERK and JNK)/NF-κB-CCL2/CXCL1 signaling pathways in cultured astrocytes, while additional studies are needed to delineate the contribution of p38 signaling pathways. TRAF6 partly expressed in microglia (6 ± 1%) except astrocytes and neurons in the study. TRAF6 is an important adaptor of the TLR4/MAPK/NF-κB signaling pathway that could induce p38/JNK phosphorylation in LPS-activated BV2 microglia cells, and TRAF6-JNK/p38-ATF2 axis might promote microglial inflammatory activation and thus aggravate neuronal injury in brain (Li et al., 2019). Inhibiting the TRAF6-related signaling pathways by alleviating microglia-mediated neuroinflammation injury could exert anti-inflammatory and neuroprotective effects (Song et al., 2016). We will establish an astrocyte-neuron co-culture and microglia-neuron co-culture to explore the role of TRAF6 signaling pathways between astrocytes/microglia and neurons in the next experiment.

TRAF6 signaling pathways mediate neuroinflammation in many diseases, so modulation of these inflammatory pathways may be a broadly effective treatment strategy. For instance, recombinant growth-arrest-specific protein 6 reduced neuroinflammation after middle cerebral artery occlusion in rats by disrupting TLR-TRAF-NF-κB signaling at the level of TRAF (Wu et al., 2018), and a small molecule inhibitor of CD40–TRAF6 signaling was shown to reduce inflammation in rodent models of multiple sclerosis (Aarts et al., 2017). Neuroinflammation was alleviated by blocking of the TRAF6/NLRP3 interaction during intracerebral hemorrhage that may shed new light on intracerebral hemorrhage treatment in clinic (Wan et al., 2021). Zinc finger protein A20 suppressed the inflammatory response following intracerebral hemorrhage by regulating TRAF6 polyubiquitination (Meng et al., 2017). MiR-146a was involved in the regulation of brain inflammation by modulating TRAF6/NF-κB or MAPK signaling pathways and could be considered a novel therapeutic agent for treating brain inflammation (Liu et al., 2020). We demonstrate that TRAF6 knockdown partially suppressed multiple inflammatory signaling pathways, reduced nerve cell apoptosis, and improved cognitive function following TBI. Therefore, further studies are warranted to explore potential therapies based on the inhibition of TRAF6-MAPKs (ERK and JNK)/NF-κB-CCL2/CXCL1 signaling following TBI.

## Conclusion

Our study demonstrates that TRAF6-MAPKs/NF-κB-CCL2/CXCL1 signaling pathways possibly mediate neuroinflammation following TBI, which in turn leads to neurodegeneration and cognitive dysfunction. Targeting these signaling pathways may provide a novel therapeutic approach for the treatment of secondary injury after TBI.

## Data Availability Statement

All data used during the current study are available from the corresponding author on reasonable request.

## Ethics Statement

The animal study was reviewed and approved by the Experimental Animal Center of Nantong University (permission number: 20191106-003). Written informed consent was obtained from the owners for the participation of their animals in this study.

## Author Contributions

HH, AX, SL, CL, YL, LS, ZZ, and SW performed the experiments. SL conceived and designed the study. JC and XZ analyzed the data. All authors read and approved the final manuscript.

## Conflict of Interest

The authors declare that the research was conducted in the absence of any commercial or financial relationships that could be construed as a potential conflict of interest.

## Abbreviation

AAV: adeno-associated virus
AAV9: adeno-associated virus serotype 9
CCI: controlled cortical impact
CCL2: chemokine C-C motif ligand
CCR2: chemokine C-C motif receptor 2
CNS: central nervous system
CXCL1: chemokine (C-X-C motif) ligand 1
CXCR2: chemokine C-C motif receptor 2
DAPI: 4′,6-diamidino-2-phenylindole
DMSO: dimethyl sulfoxide
ELISA: enzyme-linked immunosorbent assay
ERK: extracellular signal-regulated kinase
JNK: c-Jun N-terminal kinase
LPS: lipopolysaccharide
MAPKs: mitogen-activated protein kinases
NC: negative control
NF-κB: nuclear factor-kappa B
siRNA: small interfering ribonucleic acid
sTBI: severe traumatic brain injury
TBI: traumatic brain injury
TRAF6: TNF receptor-associated factor 6
TUNEL: terminal deoxynucleotidyl transferase-mediated dUTP nick-end labeling.
